# Integrated Transcriptome and Metabolome Analyses Reveal Bamboo Culm Color Formation Mechanisms Involved in Anthocyanin Biosynthetic in *Phyllostachys nigra*

**DOI:** 10.3390/ijms25031738

**Published:** 2024-02-01

**Authors:** Ou Cai, Hanjiao Zhang, Lu Yang, Hongyu Wu, Min Qin, Wenjing Yao, Feiyi Huang, Long Li, Shuyan Lin

**Affiliations:** 1 Co-Innovation Center for Sustainable Forestry in Southern China, Nanjing Forestry University, Nanjing 210037, China; caiou199913@163.com (O.C.); 18952299369@139.com (H.Z.); 15519737210@163.com (L.Y.); why05292021@163.com (H.W.); mq178828@163.com (M.Q.); yaowenjing@njfu.edu.cn (W.Y.); hfy@njfu.edu.cn (F.H.); 2 Bamboo Research Institute, Nanjing Forestry University, Nanjing 210037, China; 3 College of Life Sciences, Nanjing Forestry University, Nanjing 210037, China

**Keywords:** *Phyllostachys nigra*, anthocyanin, culm color, MYB, transcriptome and metabolome

## Abstract

*Phyllostachys nigra* has green young culms (S1) and purple black mature culms (S4). Anthocyanins are the principal pigment responsible for color presentation in ornamental plants. We employ a multi-omics approach to investigate the regulatory mechanisms of anthocyanins in *Ph. nigra*. Firstly, we found that the pigments of the culm of *Ph. nigra* accumulated only in one to four layers of cells below the epidermis. The levels of total anthocyanins and total flavonoids gradually increased during the process of bamboo culm color formation. Metabolomics analysis indicated that the predominant pigment metabolites observed were petunidin 3-*O*-glucoside and malvidin *O*-hexoside, exhibiting a significant increase of up to 9.36-fold and 13.23-fold, respectively, during pigmentation of *Ph. nigra* culm. Transcriptomics sequencing has revealed that genes involved in flavonoid biosynthesis, phenylpropanoid biosynthesis, and starch and sucrose metabolism pathways were significantly enriched, leading to color formation. A total of 62 differentially expressed structural genes associated with anthocyanin synthesis were identified. Notably, *PnANS2*, *PnUFGT2*, *PnCHI2*, and *PnCHS1* showed significant correlations with anthocyanin metabolites. Additionally, certain transcription factors such as PnMYB6 and PnMYB1 showed significant positive or negative correlations with anthocyanins. With the accumulation of sucrose, the expression of PnMYB6 is enhanced, which in turn triggers the expression of anthocyanin biosynthesis genes. Based on these findings, we propose that these key genes primarily regulate the anthocyanin synthesis pathway in the culm and contribute to the accumulation of anthocyanin, ultimately resulting in the purple-black coloration of *Ph. nigra*.

## 1. Introduction

*Ph. nigra* is an important ornamental bamboo species in the genus *Phyllostachys*, with young culms that are light green, then gradually develop purple spots, and finally become purple-black. The presentation of plant color is mainly caused by a combination of various pigments, of which anthocyanins have a significant impact on plant coloration and are important products of flavonoid metabolism [1]. In nature, anthocyanins have six main types: cyanidin, delphinidin, pelargonidin, peonidin, petunidin, and malvidin [2]. Anthocyanins are mainly found in the vesicles of plant cells, which can not only make the plant orange, red, purple, blue, and other colors, but also protects the plant from bacteria and pests and other biological stress [2,3]. Therefore, anthocyanins have diverse functions and have garnered significant attention as a research hotspot in the field of secondary metabolites of ornamental plants. Anthocyanin biosynthesis is controlled by multiple structural enzymes, including chalcone synthetase (CHS), chalcone isomerase (CHI), and flavanone 3-hydroxylase (F3H), dihydroflavonol 4-reductase (DFR), and anthocyanin synthase (ANS) [3,4]. The first phase is the production of coumaroyl-coA, which is a common pathway in most plant secondary metabolisms. The second stage is the generation of dihydrokaempferol from coumaroyl-coA catalyzed by CHS, CHI, and F3H, which is a key step in the plant flavonoid synthesis pathway. These two stages are critical steps in the formation of substrates for the anthocyanin synthesis pathway and are the basis of the anthocyanin metabolic pathway. Flavonoid 3′-hydroxylase (F3′H) and flavonoid 3′ 5′-hydroxylase (F3′5′H) catalyze the hydroxylation of dihydrokaempferol to form dihydroquercetin and dihydromyricetin. The third stage is the most important stage of anthocyanin metabolism. Dihydroflavonols can be converted to colored anthocyanins by DFR and ANS. The oxidation reaction of *ANS* is one of the central steps in the anthocyanin pathway [5,6,7]. In order for anthocyanin to be stabilized within the plant, it needs to be formed into anthocyanin glycosides by the enzyme flavonoid 3-*O*-glucosyl transferase (UFGT). *O*-methyltransferase (OMT*)* is one of the key enzymes for anthocyanin modification and flower pigmentation that results in the formation of other types of anthocyanins [8,9]. Therefore, loss of function or low expression of *ANS*, *UFGT*, and *OMT* results in the loss or reduced accumulation of anthocyanins, which affects plant coloration [9,10]. *DoANS* and *DoUFGT* are key regulatory genes associated with the differential accumulation of anthocyanin in Dendrobium (*Dendrobium officinale*) culms [11].

Structural genes associated with anthocyanin biosynthesis are regulated by transcription factors, including those of the MYB, bHLH, and WD40 gene families [2,12]. The plant MYB family is one of the most important families of transcription factors that regulate specific processes such as anthocyanin biosynthesis, signal transduction, environmental stress, and disease resistance [13]. In *Arabidopsis*, the overexpression of MYB transcription factors and WD40 proteins activates the anthocyanin pathway in leaves and stems, leading to a high accumulation of anthocyanins in plants [14]. Studies have shown that MYB transcription factors can regulate anthocyanin formation in conjunction with protein complexes formed by bHLH and WD40 [15,16]. In *Gerbera hybrida* [17] and *Cinnamomum camphora* [18]*,* the MYB and bHLH drive other anthocyanin synthesis-related genes to regulate anthocyanin synthesis in leaves, stems, and flowers. Up-regulation of MYB in the red stalks of *Prunus mume* ‘Purpurea’ may be a crucial factor in altering anthocyanin biosynthesis, ultimately affecting the formation of red coloration in the stalk [19]. In addition, the production of anthocyanins is stimulated by sucrose-specific signaling pathways, which have been shown in *Arabidopsis thaliana* [20,21,22]. In addition, some other transcription factors may also affect anthocyanin biosynthesis. For example, AtERF4 and AtERF8 contribute to light-modulated anthocyanin biosynthesis in *A. thaliana* [23]. WRKY has been reported to be involved in anthocyanin biosynthesis in *A. thaliana* [24] and *Rhododendron simsii* [25]. bZIP also plays a key role in anthocyanin synthesis [26,27].

In this study, we investigated the metabolite differences in the culm of *Ph. nigra* at four color formation periods using liquid chromatography-mass spectrometry (LC-MS). Based on comparison analysis of transcriptome sequencing data, potential genes responsible for anthocyanin synthesis were screened and further verified by real-time quantitative polymerase chain reaction (qRT-PCR). The present findings provide insight into the regulatory mechanism of these genes in the anthocyanin metabolic pathway and theoretical support for the advancement of molecular breeding in bamboo ornamental plants.

## 2. Results

### 2.1. Phenotypic Analysis of Ph. Nigra of Culm Different Developmental Stages

**Figure 1:**
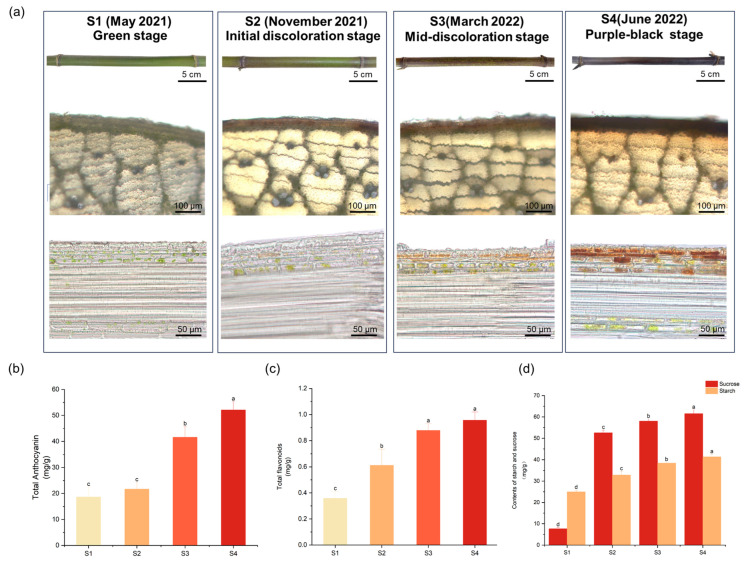
Anthocyanins and flavonoids accumulated in culms of *Ph. nigra.* (**a**) Phenotypes of the culm and images of transverse sections in *Ph. nigra*. The second row is a cross-section and the third row is a longitudinal section. May 2021, November 2021, March 2022, and June 2022 represent the time of sample collection at each stage. (**b**) The total anthocyanin content of culms. (**c**) The total flavonoid content of culms. (**d**) Contents of starch and sucrose. Different letters above the bars indicate a significant difference (one-way ANOVA: *p*  <  0.05).

We conducted dynamic monitoring of the color changes of *Ph. nigra* culms from May 2021 to June 2022. Based on the observed dynamic changes in the culm coloring process, we selected four represented stages, including the green stage (S1), the stage in which culm pigmentation spots began to appear (S2), the stage in which culm pigmentation spots appeared in large quantities (S3), and the stage in which the culm was purple-black (S4), for further anatomical analysis. Observation of the transverse sections of culms showed that *Ph. nigra* exhibited aggregates of pigment in one to four layers of cells below the epidermis (Figure 1a). As *Ph. nigra* developed, the total levels of anthocyanins showed an upward trend. Even in the green stage, although there were small amounts of anthocyanins in the culm, it still exhibited a green phenotype (Figure 1b). Likewise, the total flavonoid content showed a gradual increase, following a pattern similar to that of the total anthocyanin content (Figure 1c). Furthermore, the content of sucrose and starch exhibited a gradual increase throughout the four stages, with significant differences between them (Figure 1d).

### 2.2. Metabolome Profiling of Culms of Different Stages

**Figure 2:**
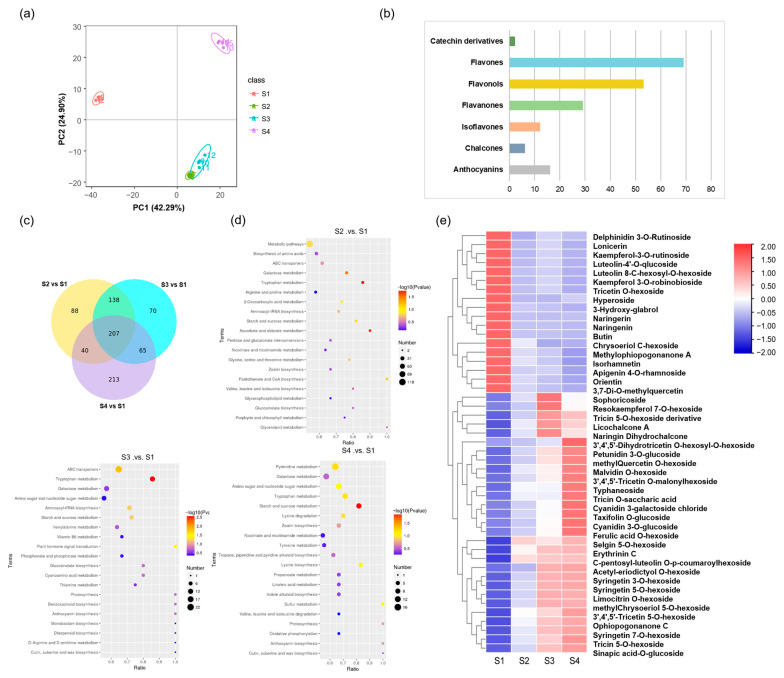
Differentially accumulated metabolites analysis of metabolome. (**a**) PCA score plot. (**b**) Detailed classification of flavonoid metabolites. (**c**) Venn diagram of DAMs. (**d**) KEGG enrichment of DAMs in each comparison group. (**e**) The heatmaps of DAMs are in S4-vs.-S1 and S3-vs.-S1.

In order to further clarify which components were the decisive factors for *Ph. nigra* culm color formation, a metabolome profile was carried out using ultra-high performance liquid chromatography-tandem mass spectrometry (UPLC-MS). Replicates of each sample were grouped in principal component analysis (PCA), highlighting the quality and reproducibility of the subject datasets. Moreover, in PCA, we found that there were significant differences between S1 and the other three periods, while there were minimal changes between the S2 and S3 periods (Figure 2a). We characterized 1223 metabolites in *Ph. nigra*, including 187 flavonoids, 80 carbohydrates, 186 amino acids, and others. Among the identified flavonoids, there were 16 anthocyanins, 6 chalcones, 1 isoflavone, 29 flavanones, 53 flavonols, 69 flavones, and 2 catechin derivatives (Figure 2b). To pinpoint the significant differentially accumulated metabolites (DAMs) associated with phenotype, the variable important in projection (VIP) ≥ 1.0 together with fold change (FC) ≥ 2 or ≤ 0.5 were set as the thresholds. By comparing the metabolic profiles of four samples, we found that there were 473 DAMs in the S2-vs.-S1 comparison, 480 DAMs in the S3-vs.-S1 comparison, and 525 DAMs in the S4-vs.-S1 comparison. (Figure 2c). Among these, 207 metabolites showed differential accumulation in all three comparative groups. The Kyoto Encyclopedia of Genes and Genomes (KEGG) annotation of DAMs revealed their association with anthocyanin biosynthesis, indicating that the purple-black color of *Ph. nigra* culms is primarily attributed to anthocyanins (Figure 2d). However, the anthocyanin synthesis pathway did not show significant enrichment in the S2-vs.-S1 comparison. Instead, pathways such as ascorbate and aldarate metabolism, tryptophan metabolism, and galactose metabolism were more prominently enriched. The starch and sucrose synthesis pathways were significantly enriched with metabolites in all three comparison groups, and sucrose content in the metabolic group was significantly increased by 450-fold, 727-fold, and 880-fold, respectively (Table S1).

In S3-vs.-S1 and S4-vs.-S1, we identified 45 differentially accumulated flavonoids (DAFs) in *Ph. nigra* (Table S1). These DAFs were analyzed to further explore the reasons why the culms appear purple-black. The concentration levels of petunidin 3-*O*-glucoside (Ptgl), malvidin *O*-hexoside (Mvhx), cyanidin *O*-syringic acid (Cysy), cyanidin 3-*O*-glucoside (Cygl), and cyanidin 3-galactoside chloride (Cycl) exhibited a gradual increase trend (Figure 2e). Of particularly note, petunidin 3-*O*-glucoside and malvidin *O*-hexoside exhibited significant differences in all three comparison groups, with an increase of up to 9.36-fold and 13.36-fold, respectively. In addition, many flavonoids, flavonols, isoflavonoids, and other DAFs were gradually increased, including syringetin 7-*O*-hexoside with an increase of up to 80-fold, indicating that flavonoids may also play an important role in culm color formation of *Ph. nigra* (Table S1). We also found significantly higher levels of the lignin-related metabolites sinapic acid-*O*-glucoside and ferulic acid *O*-hexoside, suggesting not only the accumulation of anthocyanins but also the increase of lignin content during the growth of *Ph. nigra* (Table S1; Figure S1). However, there were two differential anthocyanins in S3-vs.-S2 and S4-vs.-S3, pelargonidin o-acetylhexose and pelargonidin 3-o-propylene glycol hexose, but their metabolic levels showed a downward trend in the S4 stage (Figure S2).

### 2.3. Transcriptome Analysis and Differential Expressed Gene Identification of Different Stages of Ph. nigra

**Figure 3:**
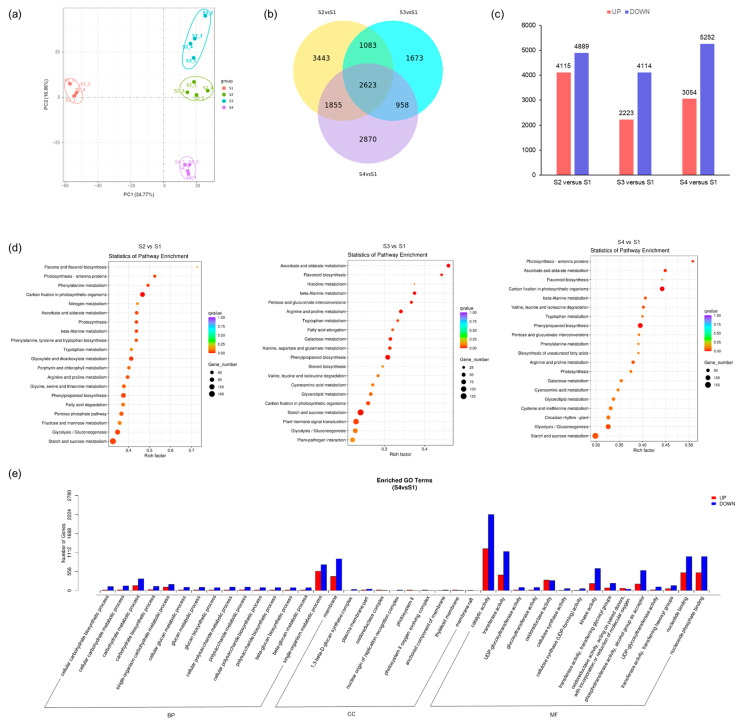
DEG analysis in different stages of *Ph.nigra.* (**a**) PCA score plot. (**b**) Venn diagram of DEGs. (**c**) The number of differentially expressed genes in three comparison groups. (**d**) KEGG enrichment of DEGs in each comparison group. The *q* value ranges from [0–1]. The closer that number is to 0, the more significant the enrichment is. The greater the rich factor, the greater the degree of enrichment is. (**e**) GO enrichment analysis of DEGs between S4 and S1. The *q*-value is the multiple hypothesis test-corrected *p* value.

Transcriptomic analysis was performed to understand the genetic mechanism of color formation in *Ph. nigra* culms. The PCA demonstrated the reproducibility of gene expression patterns across different stages of *Ph. nigra*, as observed in the four biological replicates (Figure 3a). In the comparison of differentially expressed genes (DEGs) from different stages of *Ph. nigra*, 2623 genes were found to be differentially expressed in the three comparison groups. A total of 9006, 6337, and 8306 DEGs were identified in the pairwise comparisons of S2-vs.-S1, S3-vs.-S1, and S4-vs.-S1, respectively (Figure 3b).

The S4-vs.-S1 combination had 3054 upregulated and 5252 downregulated genes in *Ph. nigra* (Figure 3c). To identify the DEGs that are involved in metabolic pathways in different stages, Gene Ontology (GO) and KEGG enrichment analyses were subjected to all the DEGs (Figure 3d,e). In all comparison groups, GO term analysis revealed that DEGs were enriched in catalytic and transferase activities. The KEGG showed high enrichment of DEGs in the flavonoid biosynthesis and flavone and flavonol biosynthesis pathways; additionally, a significant number of DEGs were enriched in the phenylpropanoid biosynthesis, starch, and sucrose metabolism pathways. It is evident from the KEGG analysis that the DEGs are significantly associated with various metabolic pathways, including flavonoid biosynthesis, flavone and flavonol biosynthesis, starch and sucrose metabolism, and phenylpropanoid biosynthesis, among others.

### 2.4. Candidate Genes Related to the Anthocyanin Biosynthesis Pathway

**Figure 4:**
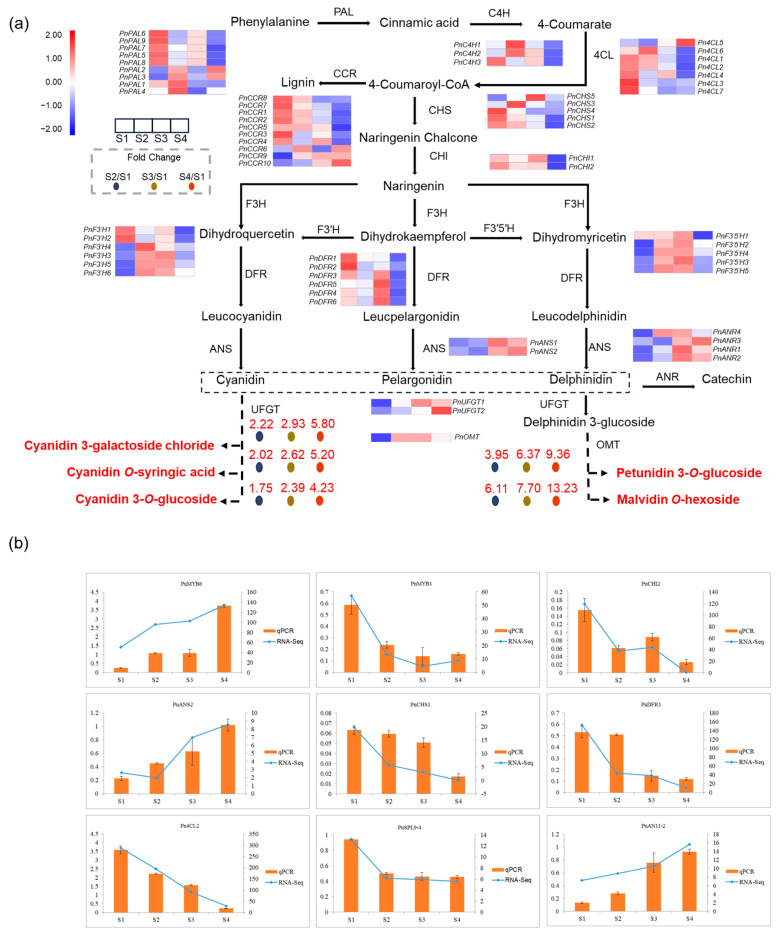
Anthocyanin biosynthetic pathway and their gene expression levels in *Ph.nigra.* (**a**) DEGs involved in anthocyanin biosynthesis. Blue and red in the heatmap represent the down- and up-regulated structural genes, respectively. Differentially expressed genes including phenylalanine ammonia-lyase (*PAL*), cinnamate 4-monooxygenase (*C4H*), 4-coumarate-CoA ligase (*4CL*), cinnamoyl-Co A reductase(*CCR*), chalcone synthase (*CHS*), chalcone isomerase (*CHI*), flavanone-3-hydroxylase (*F3H*), flavonoid 3′-hydroxylase (*F3′H*), flavonoid 3′,5′-hydroxylase (*F3′5′H*), dihydroflavonol 4-reductase (*DFR*), anthocyanidin reductase (*ANR*), anthocyanidin synthase (*ANS*) and UDP-glucose: flavonoid-3-*O*-glycosyltranferase (*UFGT*), *O*-methyltransferase (*OMT*). Four colored boxes show the relative gene expression level measured by RNA-seq (average value). (**b**) Relative expression of selected genes validated by qPCR. Error bars indicate standard deviation (SD) of three technical repeats.

Previous work has demonstrated the crucial role of the anthocyanin biosynthetic pathway as a key branch within the flavonoid pathway responsible for the synthesis and production of anthocyanins. Therefore, to further explore the mechanisms underlying purple-black pigmentation in *Ph. nigra* culms, the expression patterns of genes involved in anthocyanin biosynthetic pathways were analyzed based on a comparative transcriptome analysis. The flavonoid biosynthesis pathway was mapped, and a total of 62 DEGs involved in the flavonoid pathway were identified from the comparative analysis of S2-vs.-S1, S3-vs.-S1, and S4-vs.-S1, including phenylalanine ammonia-lyase (*PAL*), cinnamate 4-monooxygenase (*C4H*), 4-coumarate-CoA ligase (*4CL*), cinnamoyl-Co A reductase(*CCR*), anthocyanidin reductase (*ANR*), *CHS*, *CHI*, *F3′H*, *F3′5′H*, *DFR*, *ANS*, *UFGT,* and *OMT* (Figure 4a). Among these genes, a total of 23 exhibited significant differential expression across all three comparative groups (Table S2). Based on our transcriptome data analysis, most of the candidates showed high transcriptional activity during at least one stage of culm color formation.

When comparing to S1, the *PnPAL* and *PnC4H* genes exhibited a significant decrease in expression in S4. The *Pn4CL* genes exhibited significant differences in expression levels, including one up-regulated and six down-regulated genes. The CHS and CHI are the key enzymes in the upstream of the flavonoid synthesis pathway. Their expression consistently exhibited a gradual decrease over the four stages of culm color formation. Additionally, the content of naringenin chalcone and naringenin was higher during the green stages and displayed a gradual decrease (Table S1). The *PnCCRs* exhibit two distinct expression modes, with either a continuous increase or a continuous decrease in expression levels. This suggests that they may have different functions during specific stages of culm color formation in *Ph. nigra*. On the other hand, the variations observed in the expression of *PnDFRs*, including both increases and decreases, may be attributed to the influence of certain regulatory factors. The trend for *PnF3’H* and *PnF3’5’H* was initially growing and then declining, but the S4 expression level was higher than the S1 (Table S2). The expression levels of downstream structural genes, including *PnANS*, *PnANR*, *PnUFGT,* and *PnOMT*, were significantly upregulated during the late color formation stage of culm.

In short, the expression pattern of these structural genes likely contributes to the purple-black coloring of *Ph. nigra*. The results showed that the expression trend of differentially accumulated flavonoids was consistent with the associated genes. To ensure the reliability of the transcriptome sequencing data, we conducted qRT-PCR detection on essential genes (Table S3). The results of qRT-PCR were in agreement with the transcriptome sequencing analysis, suggesting the reliability of transcriptome analysis for identifying DEGs (Figure 4b).

### 2.5. Transcription Factor Profiling and Characterization

**Figure 5:**
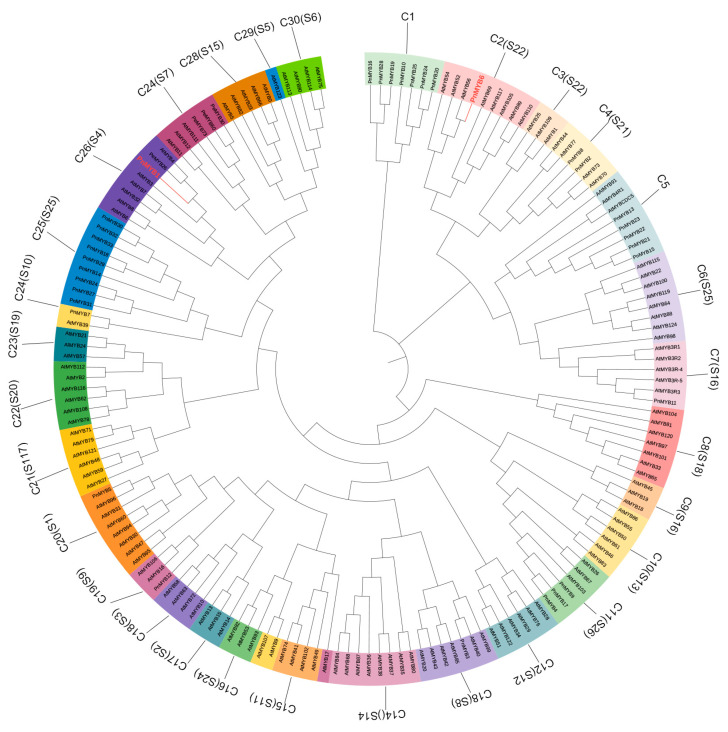
Phylogenetic tree of *MYB* of *Ph. nigra* with *MYBs* of *Arabidopsis* and moso bamboo.

Transcription factors play an important role in the regulation of anthocyanin biosynthesis by regulating the expression level of structural genes. MYB, bHLH, and WD40 are the three most important families of transcription factors in the regulation of anthocyanin synthesis. A total of 33 MYBs, 39 bHLHs, 3 WD40 proteins, and 4 SPL9s were differentially expressed (Table S2). To predict the functions of these TFs, the phylogenetic trees were constructed. The *A. thaliana* 131 members of the MYB family and 161 members of the bHLH family and 4 MYBs (PeMYB30, PeMYB26, PeMYB60, PeMYB73) of *Ph. edulis* associated with anthocyanins were used (Figure 5). Phylogenetic analysis indicated that PnMYB1 belongs to the C26 (*Arabidopsis* S4 subgroups) of the clade. S4 subgroups of *A. thaliana* include repressor MYBs involved in the regulation of anthocyanins and flavonols biosynthesis [28]. PnMYB6 belongs to clade C2 and is clustered with AtMYB56. AtMYB56 regulates the level of free maltose and the subsequent accumulation of anthocyanins in plants [29]. The content of sucrose also increased gradually in the metabolome (Table S1). The bHLHs of the branches of PnbHLH23 and PnbHLH27 both act as transcriptional inhibitors to negatively regulate anthocyanin biosynthesis [30,31] (Figure S3). Therefore, based on phylogenetic tree analysis, it can be inferred that the aforementioned transcription factors have functions similar to those in *A. thaliana* in regulating anthocyanin synthesis in *Ph. nigra*.

In addition, three AN11s and four SPL9s were identified in the annotation library. AN11 is a WD40 protein that not only forms MBW complexes with bHLH and MYB to activate anthocyanin-related genes but also regulates anthocyanin synthesis independently [32]. The expression levels of the three PnAN11s were gradually increased in *Ph. nigra* culms (Table S2). It is reported that SPL TF is an anthocyanin synthesis inhibitor [33]. In the transcriptome and metabolome, the expression of SPL9 gradually decreased, but the anthocyanin content gradually increased (Table S2). Therefore, we suggested that PnMYB1, PnMYB6, PnbHLH27, PnbHLH32, PnAN11s, and PnSPL9s play crucial roles as regulators in the anthocyanin biosynthesis pathway. We also screened 27 AP2/ERF, 17 WRKY, and 27 bZIP in the DEGs (Table S2). It has been well documented that these transcription factors are involved in regulating the synthesis of anthocyanins.

### 2.6. Co-Expression Analysis of Genes Related to the Anthocyanidin Biosynthesis Pathway

**Figure 6:**
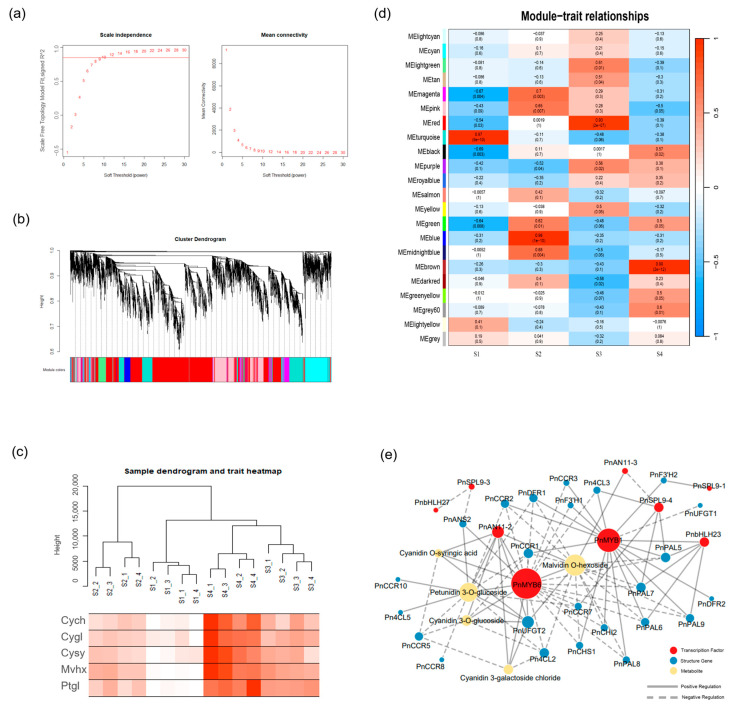
Co-expression network analysis. (**a**) Soft threshold. (**b**) Hierarchical cluster tree obtained by weighted gene co-expression network analysis (WGCNA). (**c**) Sample dendrogram and trait heatmap. petunidin 3-*O*-glucoside (Ptgl), malvidin *O*-hexoside (Mvhx), cyanidin *O*-syringic acid (Cysy), cyanidin 3-*O*-glucoside (Cygl), and cyanidin 3-galactoside chloride (Cycl). (**d**) Matrix of module-stages associations. The gray modules represent genes that are not divided into specific modules. (**e**) Co-expression networks of differentially expressed genes and anthocyanins metabolites associated.

To identify genes associated with color variations in purple bamboo more comprehensively, weighted gene co-expression network (WGCNA) analysis was performed on all expressed genes. When the growth period cluster map was generated, it was observed that there was a consistent upward trend in the correlation between all five anthocyanin metabolites and the color intensity (Figure 6c). All genes were clustered into 22 modules, with the brown and turquoise modules being the most closely related to S4 and S1, respectively (Figure 6d). Total anthocyanins, total flavonoids, and anthocyanin metabolites exhibited a significant positive correlation with the brown module while showing a negative association with the turquoise module (Figure S4), indicating that the genes within the brown and sapphire blue modules primarily contribute to the coloration of *Ph. nigra*. We identified 36 genes associated with anthocyanin synthesis in these two modules, including PnMYB6, PnMYB1, PnbHLHLs, PnAN11s, *PnANSs*, *PnANRs*, *PnF3′H1, PnF3’5’H1*, *PnDFRs*, *PnCHIs*, *PnCHSs*, and others (Table S4). To identify hub genes within the modules, we employed module eigengene-based connectivity (kME) analysis using WGCNA. In addition to *PnMYB1*, *PnCHS1*, *PnDFR1*, *Pn4CL3, PnCCR1*, and *PnCCR2*, we used a threshold screening approach (kME > 0.9) and discovered seven AP2/ERF-ERF, two bZIP, two WRKY*,* and six ABC transporters (Table S4). All these genes have been reported to be involved in the regulation of anthocyanin and flavonoid biosynthesis. Particularly, the ABC transporters are primarily responsible for transferring anthocyanins into vesicles during anthocyanin.

The regulatory network map was constructed by calculating the Pearson correlation coefficient (PCC), which depicts the relationship between the key metabolites and genes (Figure 6e). We found that the genes that were significantly positively correlated with differentially accumulated anthocyanins (DAAs) were *PnANS2*, *Pn4CL5*, *PnUFGT2*, *PnMYB6*, *PnANN11-2*, *PnAN11-3*, and *PnCCR13* (r > 0.8 and *p* < 0.01). The *Pn4CL2*, *PnCHI2*, *PnCHS1,* and 6 *CCR* were negatively correlated with the DAAs (r < −0.8 and *p* < 0.01). Through the calculation of Pearson correlation, PnMYB6 and upstream *PnCCRs PnCHIs*, *PnCHSs*, *Pn4CLs* show significant negative correlation, but with downstream key gene *PnANSs*, *PnUFGTs,* and *PnANRs* present positive correlation. However, PnMYB1 was negatively correlated with downstream genes *PnF3′5′H*, *PnANSs*, *PnUFGT1,* and *PnOMT* (Table S5). Moreover, PnMYB6 and PnMYB1 are hub genes in the regulatory network which play a decisive role in the culm color formation of *Ph. nigra*, and the regulatory relationship needs to be further studied (Figure 6e).

## 3. Discussion

Plant colors are affected by anthocyanin accumulation. In sweet osmanthus, anthocyanin accumulation was significantly correlated with pericarp color [34]. Anthocyanin contents were significantly increased with the development of purple wheat (*Triticum aestivum* L.) [35]. The difference in color between the apical and basal parts of *Petunia* petals is due to the difference in anthocyanin content [36]. Our study aims to elucidate the role of anthocyanins in the pigmentation of *Ph. nigra* culms. We analyzed the accumulation of anthocyanin metabolites and genes involved in anthocyanin synthesis pathways to gain insights into this process. Anthocyanins are pigments commonly found in plant epidermal cells, with some also present in the mesophyll layer [1,37]. In our study, we observed that anthocyanins were exclusively located in one to four layers of cells below the epidermis of *Ph. nigra* culms. Furthermore, we found that during the color formation stage, not only did the content of anthocyanins and flavonoids gradually increase, but sucrose and lignin were also undergoing similar changes. Through our investigation, we identified two primary anthocyanins responsible for the pigmentation in *Ph. nigra* culms: petunidin 3-*O*-glucoside and malvidin *O*-hexoside. During S4, their metabolic levels were as high as 1548,000 and 433,175, respectively. These two anthocyanins give the culms a blue to purple hue, which is commonly observed in nature [2].

The expression of *CCR* and *CHS* genes in phenylpropanoid biosynthesis directly affects the direction of substrate conversion and the downstream metabolic march of phenylalanine metabolism. *CCR* expression can affect the lignin content in plants, which is a crucial component of bamboo plants, and the lignin content rises with the age of bamboo [38,39,40]. The regulation of *CCR* is complex and requires further study because of the different expression patterns of *CCR* genes and the gradual increase in the content of both anthocyanins and lignins in *Ph. nigra.*

Differences in plant anthocyanin composition arise from variations in gene expression within the anthocyanin synthesis pathway. The expression levels of *PnCHS* and *PnCHI* peaked at S1, then declined and became almost undetectable at S4. Furthermore, they play a role in the production of compounds such as naringenin chalcone and naringenin, and these compounds exhibit metabolomic patterns that correspond to the levels of gene expression. It is possible that upstream genes such as *PnPALs*, *PnCHSs*, *PnCHIs* in each period were regulated by feedback repression of their own products, which suppressed their expression in later stages. And it has also been reported that there are feedback inhibitory effects in the flavonoid synthesis pathway with complexity and diversity [41,42].

The two branches of anthocyanin biosynthesis are regulated by *F3’H* and *F3’5’H*, respectively, affecting the anthocyanins’ color and composition in the bamboo culms. Since their expression levels are highest during S2 and S3, it may be concluded that these two periods are the crucial times for *Ph. nigra* color alteration. ANS is the primary enzyme responsible for synthesizing pigmented anthocyanins, and it exhibited a notable up-regulation trend during the S2, S3, and S4 periods. The co-expression network analysis revealed a positive correlation between the expression trend of *PnANS2* and that of DAAs, indicating the crucial role of *PnANS2* in anthocyanin synthesis. Furthermore, it is worth noting that glycosylation plays a critical role in stabilizing unstable anthocyanins and aglycones, as well as serving as a signal for anthocyanin transport into the vacuoles. This ultimately leads to the accumulation of anthocyanins in plants, and UFGT plays an important role in this process [43]. In this study, *PnUFGT* exhibited a high expression during the S2 to S4 period, coinciding with the onset of color development in the culms. These results indicate that the high expression of *PnF3′Hs*, *PnF3′5′Hs*, *PnANSs,* and *PnUFGTs* contributes to the accumulation of substantial amounts of anthocyanins. Overall, the results of the functional annotation of the DEGs suggest that they may have a role in the color formation of *Ph. nigra,* which could be an indication of their involvement in the color formation of *Ph. nigra*.

The major regulatory factors for anthocyanins are MYB, bHLH, and WD40. These transcription factors are capable of forming the MBW complex, which has been shown to play a critical role in anthocyanin synthesis in a wide range of plant species [44,45]. The regulation of anthocyanins by MYB has been studied in *Arabidopsis* [46], *Rosa rugose* [47], *Tulipa gesneriana* [48], etc. According to the phylogenetic tree, PnMYB1 is grouped in the same subgroup as AtMYB3 and exhibits a comparable role in restricting anthocyanin synthesis by repressing phenylalanine metabolism [28]. Our study found a significant negative correlation between PnMYB1 and DAAs, indicating that PnMYB1 acts as a suppressor and inhibits the synthesis of anthocyanins. In the phylogenetic tree, PnMYB6 and AtMYB56 are located in the same branch with similar functions. AtMYB56, a transcription factor, responds to sucrose and plays a role in regulating the anthocyanin accumulation. Sucrose is not only integral to signal transduction and energy production during the plant life cycle, but also serves as a primary messenger. Sugars, in this capacity, participate in signal transduction and regulate various processes, including nutrient mobilization, photosynthesis, and flavonoid biosynthesis [49]. Sucrose has been found to regulate anthocyanin biosynthesis in the *Petunia* crowns [50], *Arabidopsis* [29] hypocotyls of *Raphanussativus* [51]. In both the metabolome and transcriptome of *Ph. nigra*, the sucrose and starch synthesis pathways showed significant enrichment of DAMs and DEGs in all three comparison groups. Based on the results, the content of sucrose increased significantly and exhibited a consistent trend with the metabolome. Sucrose metabolism levels climbed from 73,275 to 64,465,000, an 880-fold increase over S1 during the S4 period. Thus, we suggest that sucrose acts as a signal to induce anthocyanin accumulation and that PnMYB6 can act in a sucrose-dependent manner to influence culm color in *Ph. nigra* by anthocyanin accumulation.

In conclusion, we propose that the control of structural genes like *PnF3′Hs*, *PnF3′5′Hs, PnANS2*, *PnUFGTs*, *PnCHI2*, and *PnCHS1* by transcription factors like PnMYB6, PnMYB1, PnbHLHs, PnAn11, and PnSPL9 is primarily responsible for the accumulation of anthocyanins in *Ph. nigra*. According to our findings, anthocyanin biosynthesis in *Ph. nigra* culms is a complex and dynamic process influenced and regulated by various factors. To further understand this pathway, future studies should focus on investigating the specific mechanisms of action for each of these regulators.

## 4. Materials and Methods

### 4.1. Plant Material

*Ph. nigra* was collected from Nanjing Forestry University, Nanjing, Jiangsu Province, China (32°04′40″ N, 118°48′42″ W). The experimental materials were categorized into four stages (S1, S2, S3, S4) based on their color changes. S1 is the stage where the growth of young bamboo stops and the culm is green. At stage S2, purple black pigment spots begin to appear on the culm. In the subsequent S3 stage, more purple black pigment spots were observed on the culm. In S4 stage, the entire culm turns purple black. Four independent culm samples were collected at each stage and stored in a refrigerator at −80 °C for future studies.

### 4.2. Measurement of Total Anthocyanin Contents

The extraction of total anthocyanins was performed following the protocols of An et al. with minor modifications [52]. The absorbance values were measured with spectrophotometer at 530 nm, 620 nm, and 650 nm. The total anthocyanin content was calculated by the formula: OD = (OD_530_ − OD_620_) − 0.1(OD_650_ − OD_620_), anthocyanin content (mg/g =)  = OD/Eλ × V/m × 10^3^, where V represents the volume of the extract, m represents the weight, and Eλ represents anthocyanin molar absorption coefficients (Eλ = 4.62 × 10^4^). Three replicates were analyzed for each sample.

### 4.3. Measurement of Total Flavonoid Contents

The rutin solution was prepared by mixing methanol and anhydrous rutin to serve as the control solution [53]. Firstly, the sample was ground into a powder, and then 10 mL of a 70% methanol solution was added, which was placed in a hot water bath at 70 °C for 20 min. After extraction, the sample solution was centrifuged at 6000× *g* for 10 min, and the supernatant was obtained. Next, 1 mL of the sample solution, 0.5 mL of 5% NaNO_2_, and 0.5 mL of 10% ALCL_3_ were sequentially added to a 10 mL tube. After 6 min of solidification, 4 mL of 4% NaOH solution was added and the entire solution was shaken for 15 min. The sample underwent measurement of absorbance values at 510 nm with a UV-visible spectrophotometer, and standard curve calculations were obtained. The flavonoid content was determined utilizing the linear equation from the calibration curve: Y = 7.25X − 0.0093 (R^2^ = 0.998), where Y refers to the absorbance and X corresponds to the flavonoid content.

### 4.4. Measurement of Sucrose and Starch Contents

Sucrose content was determined using the resorcinol method and starch was determined colorimetrically using the anthrone method [54].

### 4.5. Metabolite Extraction and Analysis

A total of 100 mg of liquid nitrogen milled bamboo culm of *Ph. nigra* was taken in a test tube and vortexed and shaken after adding 500 μL of 80% methanol–water solution. The samples were incubated on ice for 5 min and then were centrifuged at 15,000× *g*, 4 °C for 20 min. Some of supernatant was diluted to a final concentration containing 53% methanol by LC-MS grade water. The samples were subsequently transferred to a fresh Eppendorf tube and then were centrifuged at 15,000× *g*, 4 °C for 20 min. Finally, the supernatant was injected into the LC-MS/MS system analysis [55].

The detection of the experimental samples using MRM (Multiple Reaction Monitoring) was based on the Novogene database (novoDB). These metabolites were annotated using the KEGG database (http://www.genome.jp/kegg/ accessed on 13 November 2022), HMDB database (http://www.hmdb.ca/ accessed on 13 November 2022), and Lipidmaps database (http://www.lipidmaps.org/ accessed on 13 November 2022). We applied univariate analysis (*t*-test) to calculate the statistical significance (*p*-value). The metabolites with VIP > 1 and *p*-value < 0.05 and fold change ≥ 2 or FC ≤ 0.5 were considered to be differential metabolites. The functions of these metabolites and metabolic pathways were studied using the KEGG database.

### 4.6. RNA Extraction and Library Construction

Total RNA was extracted from *Ph. nigra* culms at four different color formation stages using the RNA extraction kit (TIANGEN, Beijing, China). Each sample had four independent biological replicates. The quality and integrity of the RNA samples were assessed through agarose gel electrophoresis and NanoDrop2000 spectrophotometry (Thermo Fisher Scientific, Waltham, MA, USA). RNA sample quality testing, library construction, and sequencing were conducted by Novogene Biotechnology (https://cn.novogene.com accessed on 26 November 2022). Then, 16 cDNA libraries were sequenced on the Illumina NovaSeq 6000 platform (Thermo Fisher Scientific, Waltham, MA, USA), generating 150 bp paired-end reads. The sequencing data were quality-controlled using fastp (version 0.19.7) to remove low-quality reads. High-quality clean data were spliced with Trinity (v2.6.6) software [56] to obtain reference sequences (Ref) for subsequent analysis. The clean reads from each sample were mapped toward Ref, and the reads from each sample were filtered using RSEM (v1.2.15) software. The fragments per kilobase per million mapped fragments (FPKM) values and transcripts per million (TPM) were calculated for each gene in all the samples.

### 4.7. Analysis of Differentially Expressed Genes and Identification of Transcription Factors

The genes that had a *p*-adjust < 0.05 and|Log2 Fold change| ≥ 1 were classified as DEGs. GOseq (1.10.0) package and KOBAS (v2.0.12) software were used for GO enrichment analysis and KEGG pathway enrichment analysis of differentially expressed genes, respectively.

Functional annotation of the obtained transcripts was conducted based on the following databases, including NR (NCBI non-redundant protein sequences), the KEGG (Kyoto Encyclopedia of Genes and Genomes) database, the GO (Gene Ontology) database, Swiss-Prot (a manually annotated and reviewed protein sequence database), and Pfam (protein family).

*Arabidopsis* MYB and bHLH sequences were obtained from TAIR (The Arabidopsis Information Resource, https://www.arabidopsis.org/ accessed on 16 May 2023) (Table S6). The moso bamboo MYB, which is related to anthocyanin synthesis, was also selected and downloaded based on previous studies [57]. The protein sequences were subjected to multiple sequence comparisons using muscle, followed by phylogenetic tree construction using the Maximum Likelihood (ML) method in MEGA11 (v11.0.10) software.

### 4.8. Integrated Analysis between Metabolite Analysis and Transcriptome

The WGCNA R package (v4.3.0) software was utilized to identify hub genes associated with the anthocyanin metabolic pathway. All genes underwent analysis. For module creation, the default settings were utilized, although the minModuleSize and mergeCutHeight values were set to 30 and 0.25, respectively. The correlation assessment between the modules and selected samples was established with eigengene-based connectivity (kME) values. Metabolite and gene correlations associated with anthocyanin synthesis were computed using the Pearson correlation coefficient algorithm, and network diagrams were created with Cytoscape v3.6.1.

### 4.9. Quantitative Real-Time PCR (qRT-PCR)

Total RNA isolation was performed following the protocol utilizing the RNA extraction kit (TIANGEN, Beijing, China). Quantitative real-time PCR (qRT-PCR) analysis was conducted using the StepOne real-time system (Bio-Rad, Hercules, CA, USA) with 96-well plates. *TIP41* was chosen as the internal reference gene, and qRT-PCR primers were designed utilizing the Primer Premier 5.0 software, presented in Supplementary Table S1. The expression levels of diverse genes were calculated based on the control utilizing the 2^−ΔCT^ method.

## Data Availability

All relevant data can be found within the article and its Supplementary Materials.

## Supplementary Materials

The following supporting information can be downloaded at: https://www.mdpi.com/article/10.3390/ijms25031738/s1.
