# Supplementary material for: Integrated Transcriptome and Metabolome Analyses Reveal Bamboo Culm Color Formation Mechanisms Involved in Anthocyanin Biosynthetic in Phyllostachys nigra
Source: Int J Mol Sci. 2024 Feb 1;25(3):1738. doi: 10.3390/ijms25031738 (PMC10855043; doi:10.3390/ijms25031738)
Supplement: Supplementary file 1 [file ijms-25-01738-s001.zip › Supplementary Figures.pdf]

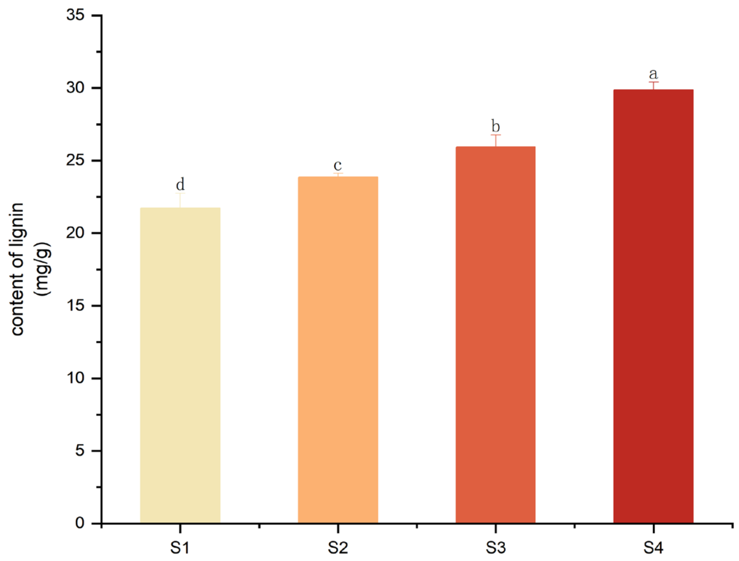


**Figure S1. Contents of lignin. Different letters above the bars indicate a significant difference (one-way ANOVA: P < 0.05).**

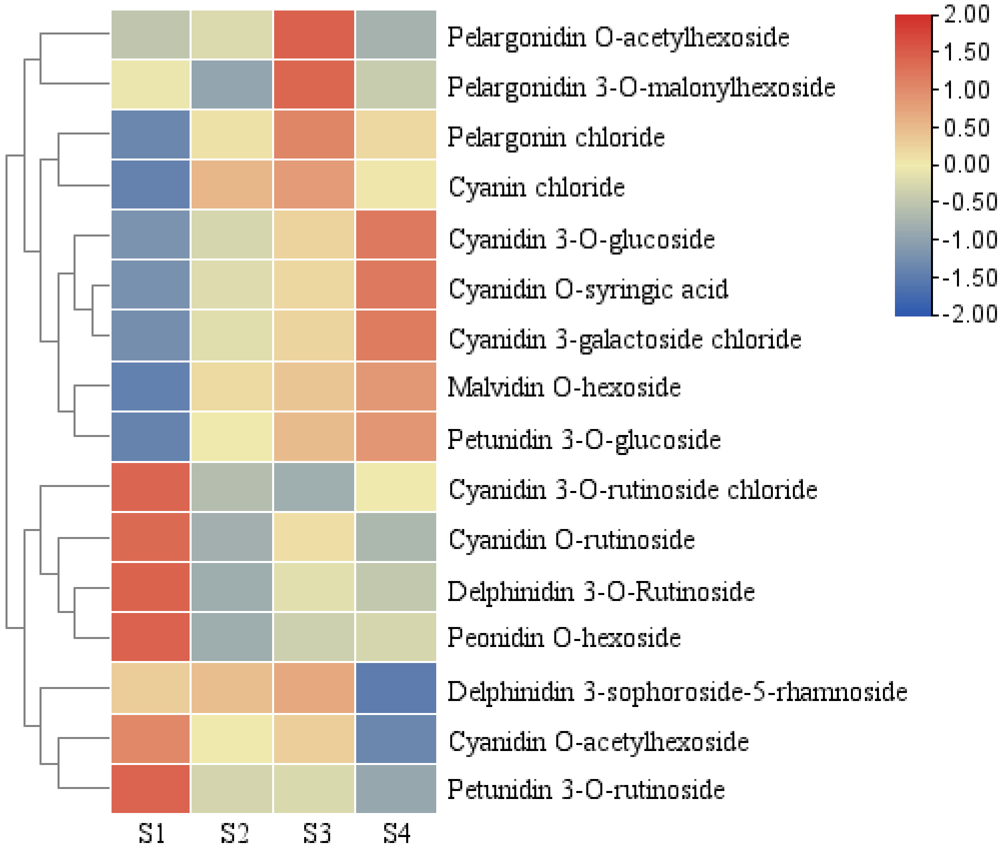


**Figure S2. The heatmaps of all anthocyanin metabolites.**

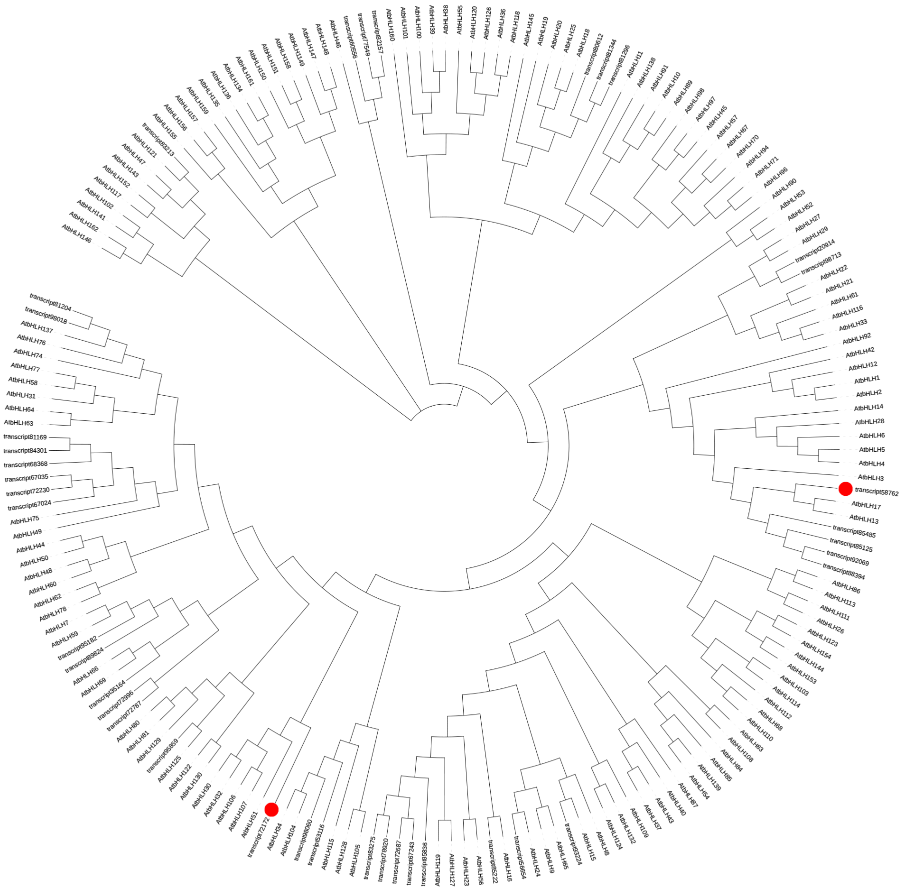

**Figure S3. Phylogenetic tree of bHLH of *Ph. nigra* with bHLHs of Arabidopsis.**

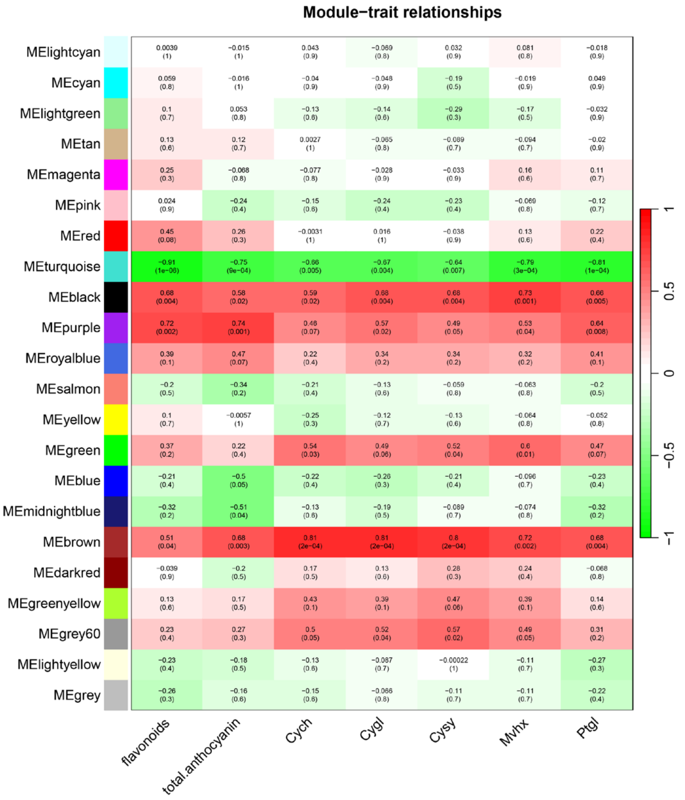


**Figure S4. Matrix of module-anthocyanins and flavonoids associations. The gray modules represent genes that are not divided into specific modules.**
